# Lifetime risks, expected years of life lost, and cost-per-life year of esophageal cancer in Taiwan

**DOI:** 10.1038/s41598-020-60701-3

**Published:** 2020-02-28

**Authors:** Wu-Wei Lai, Chia-Ni Lin, Chao-Chun Chang, Jung-Der Wang

**Affiliations:** 1 Department of Surgery, National Cheng Kung University Hospital, College of Medicine, National Cheng Kung University, Tainan, Taiwan; 2 Department of Public Health, National Cheng Kung University College of Medicine, Tainan, Taiwan; 3 Department of Internal Medicine and Occupational and Environmental Medicine, National Cheng Kung University Hospital, College of Medicine, National Cheng Kung University, Tainan, Taiwan

**Keywords:** Oesophageal cancer, Cancer epidemiology

## Abstract

Besides lifetime risks, we estimated life expectancy (LE), expected years of life lost (EYLL), lifetime costs, and cost-per-LY (life-year) stratified by different stages of esophageal cancer (EC). From the Taiwan Cancer Registry, we collected 14,420 EC to estimate the incidence rates during 2008–2014. They were followed to 2015 to obtain the survival function, which was extrapolated to lifetime by a semiparametric method. We abstracted the monthly healthcare expenditures from the reimbursement database of National Health Insurance, which were multiplied with the corresponding survival probabilities to quantify lifetime cost and cost-per-LY after adjustments. About 93.7% of patients were male; 90.8% had squamous cell carcinoma. Most patients were diagnosed at advanced stages, with 44.6% and 28.3% at stages III and IV. The lifetime risk of EC in males increased in Taiwan with a cumulative incidence rate (CIR_30–84_) of 0.0146% (2008~2010) to 0.0165% (2013–2014). The EYLL for stages I-IV were 15.8, 17.5, 20.5, and 22.5, while the average of cost-per-LY for stages I-IV were US$ 6,987, $8,776, $12,153, and $22,426. EC in Taiwan appears to have shifted into younger ages groups and incidence is still increasing. Strategies for prevention, early diagnosis and treatment are warranted to improve the cost-effectiveness and control of this cancer.

## Introduction

In Taiwan, esophageal cancer was the 9^th^ leading cause of cancer death in 2016^1–3^, which is similar to other areas of world^4^. Most esophageal cancer patients are male and diagnosed at advanced stages, which usually result in short survival and heavy financial burdens on the individual, family, and society^5^. For cancer control, stakeholders are generally interested in the following indicators: incidence rates (IR), life expectancies (LEs) after diagnosis, expected years of life lost (EYLL, i.e. loss of life expectancy), and lifetime health care expenditures^6,7^. If advanced esophageal cancer patients could be detected at an earlier stage by emerging innovations, such as novel circular RNA (CircRNAs)^8,9^, the potential benefits of reducing EYLL, lifetime cost, and productivity loss, etc. would create additional incentives for cancer screening and treatment^6,10^.

In Taiwan, the National Health Insurance (NHI) was founded in 1995, and by 2009 more than 99% of people in Taiwan were covered with full reimbursement^11,12^. All of the reimbursement-related data of the NHI have been edited and maintained by the National Health Research Institutes (NHRI) and Department of Statistics of the Ministry of Health and Welfare. Since there are relatively few studies addressing healthcare costs and outcomes from a lifetime horizon for esophageal cancer, it is worthwhile to tackle the above challenges from our nationwide large scale data stratified by stage. Through retrieving and analyzing the above national dataset, the aim of this study was to determine the lifetime risks, LEs after diagnosis, EYLL, lifetime cost, and cost-effectiveness ratio (CER) of esophageal cancer in Taiwan from 2008 to 2014 with a total number of 14,420 patients stratified by 4 stages.

## Results

**Figure 1:**
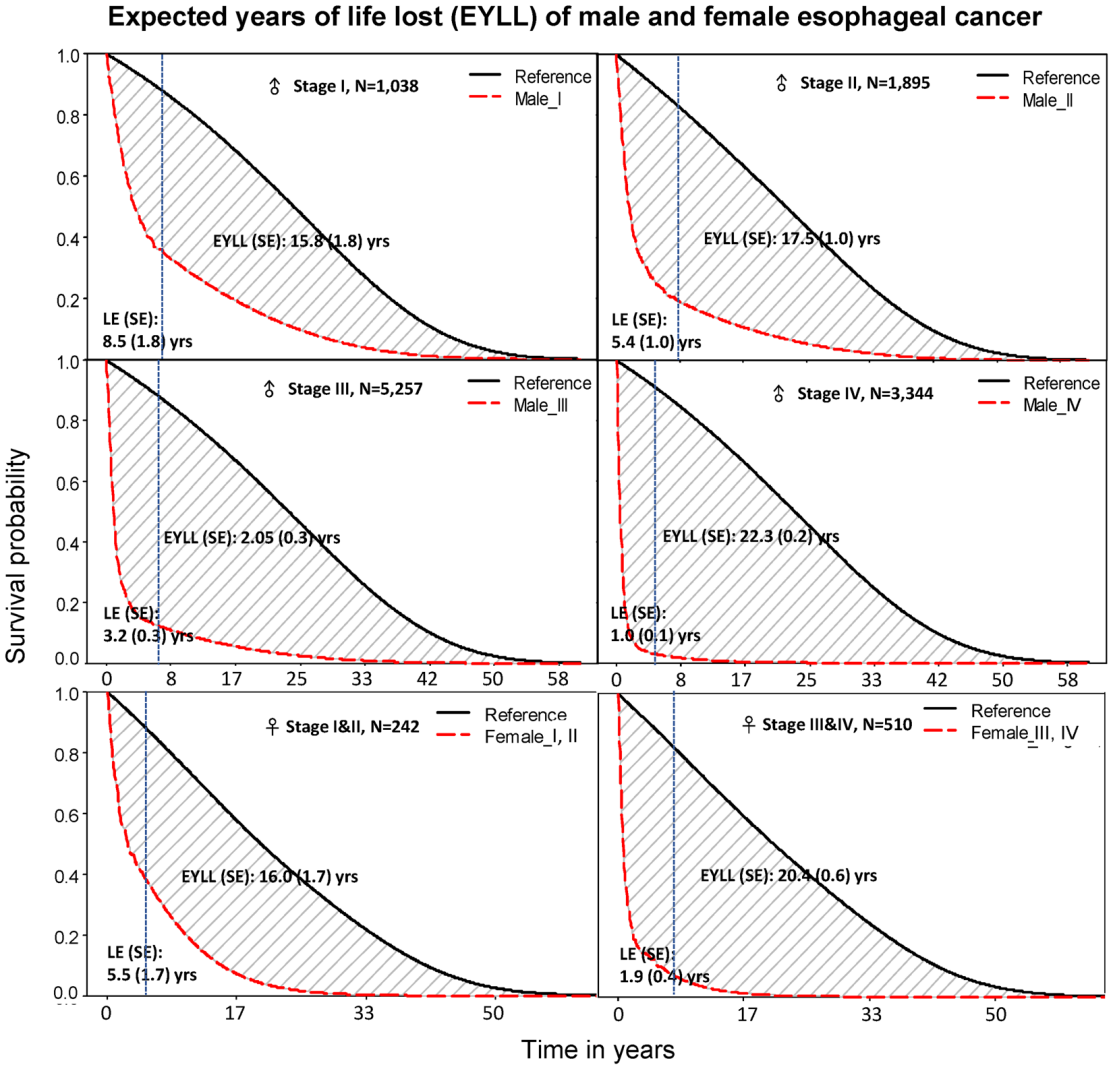
Life expectancies (LE) of esophageal cancer patients and their age- and sex-matched referents stratified by sex and stage: Shaded regions indicate the expected years of life loss (EYLL), or, loss of life expectancy that could be saved by prevention. The dotted blue line on each panel indicates the end of follow-up which is the beginning of rolling (month-by-month) extrapolation of the cancer cohort.

The mean age at diagnosis was younger for males than females (58.0 v.s. 65.2). Esophageal cancer in Taiwan is still more predominant in male (93.7%), and 90.8% are squamous cell carcinoma (Table 1).The incidence of squamous esophageal cancer in males increased gradually in Taiwan with a CIR_30–84_(‰) of 0.146 (2008~2010) to 0.165 (2013–2014) (Tables 1 and 2). A shift in the highest IR to a younger age-group from 60–69 y/o in 2008–2010 to 50–59 y/o in 2013–2014 was also noted (Table 2 and supplementary figure). Although diagnosis at earlier stages appears to have increased slightly in recent years (Table 3), most patients were diagnosed at advanced stages, with 44.6% and 28.3% at stages III and IV, respectively (Tables 1 and 3). However, the above findings were not the case for Taiwan esophageal adenocarcinoma. Table 4 summarizes the LE after diagnosis, EYLL, lifetime costs, and cost per life year of esophageal cancer. In general, the more advanced the stage, the shorter the LE and the higher the EYLL (Fig. 1). In contrast, the cost per life year of esophageal cancer increased along with advanced stage, while lifetime costs showed a decreasing trend. Patients with adenocarcinoma were diagnosed at an older age but survival was similar to those of squamous cell carcinoma, which resulted in a smaller loss-of-LE for the former and similar lifetime costs and cost-effectiveness ratios for both.

**Table 1:** Demographic characteristics of esophageal cancer patients in Taiwan, 2008–2014.

	The national cohort
Total number of patients	14,420
2008	1,657
2009	1,848
2010	2,055
2011	2,065
2012	2,171
2013	2,263
2014	2,361
**Sex, no. of patients (%)**	
Male/Female	13,515 (93.7)/905 (6.3)
**Age at diagnosis, mean (SD) years**	
Male/Female	58.0 (11.7)/65.2 (14.1)
**Age distribution, no of patients (%)**	
<50	3,471 (24.1)
50~64	6,992 (48.5)
>65	3,957 (27.4)
**Histology, no. of patients (%)**	
Squamous	13,088 (90.8)
Adenocarcinoma	476 (3.3)
Others	856 (5.9)
**Sequence of cancer diagnosed, no of patients (%)**	
1	11,803 (81.9)
>1	2,617 (18.1)
**Stage, no. of patients (%)**	
No information	1,923 (13.3)
With staging information	12,497 (86.7)
0	211 (1.7)
I	1,111 (8.9)
II	2,064 (16.5)
III	5,575 (44.6)
IV	3,536 (28.3)

**Table 2:** Incidence rate (IR, 1/10^5^ person-years) and cumulative incidence rate [CIR_30–84_(‰)] of male esophageal cancer in Taiwan stratified by histology and 5-year ages group.

Histology	Year-period	Age											
		30–34	35–39	40–44	45–49	50–54	55–59	60–64	65–69	70–74	75–79	80–84	CIR_30–84_(‰)
Sqamous cell carcinoma	2008–10	0.6	5.1	15.1	24.6	33.9	38.0	39.9	40.3	36.7	31.0	29.5	0.146
	2011–12	0.6	4.6	14.7	28.3	37.5	43.2	42.7	42.6	37.0	34.3	28.1	0.156
	2013–14	0.7	3.9	15.6	30.9	43.8	45.0	42.3	39.7	39.1	38.6	33.0	0.165
Adeno-carcinoma	2008–10	0.1	0.6	0.9	1.8	2.3	3.6	4.0	4.4	6.2	4.9	7.7	0.018
	2011–12	0.2	0.3	1.0	1.8	3.0	4.3	3.7	3.9	3.9	5.4	6.9	0.017
	2013–14	0.2	0.4	0.8	1.7	3.1	3.4	4.0	3.9	5.0	4.1	9.6	0.018

**Table 3:** Number of new cases, incident Rate (1/10^5^ person-years) and lifetime cumulative incidence rate [CIR_30–84_(1/10^5^)] of esophageal squamous cell carcinoma in males stratified by stage and period of calendar years.

		Squamous							
		Male case No.				IR (1/10^5^ person-years)			Lifetime risk
Stage	Yr period	Total	30–49	50–64	65–84	30–49	50–64	65–84	CIR_30–84_(1/10^5^)
0^*^	2008–10	41	13	16	12	0.11	0.27	0.37	0.137
	2011–12	53	21	25	7	0.28	0.55	0.32	0.204
	2013–14	68	17	38	13	0.22	0.79	0.58	0.279
I	2008–10	322	97	145	80	0.86	2.40	2.49	1.029
	2011–12	281	96	135	50	1.27	3.00	2.32	1.166
	2013–14	353	95	201	57	1.26	4.17	2.55	1.386
II	2008–10	747	180	361	206	1.59	5.98	6.41	2.494
	2011–12	485	121	249	115	1.60	5.53	5.34	2.213
	2013–14	514	133	251	130	1.76	5.21	5.81	2.293
III	2008–10	1623	493	772	358	4.35	12.80	11.14	5.006
	2011–12	1586	378	842	366	4.99	18.69	16.99	7.173
	2013–14	1704	418	931	355	5.53	19.32	15.87	7.153
IV	2008–10	1441	385	714	342	3.40	11.84	10.64	4.573
	2011–12	718	180	391	147	2.37	8.68	6.82	3.137
	2013–14	775	166	440	169	2.20	9.13	7.56	3.315

*Stage 0: T_is_ N_0_ M_0_; T_is_: carcinoma *in situ*.

**Table 4:** The LE (life expectancy), EYLL (expected years of life lost), Lifetime healthcare expenditures (in US dollars) and cost-per-life year of esophageal cancer stratified by sex, stage, and histology.

Category	stage	Onset age mean ± SD years	LE, mean (SE) years	EYLL mean (SE) years	lifetime costs (SE) [3% discount, USD]^a^	CER, Cost/LE (SE) [USD]
Male	Stage I (n = 1,038)	56.9 ± 11.3	8.5 (1.8)	15.8 (1.8)	52,096 (4,153)	6,987 (551)
	Stage II (n = 1,895)	58.7 ± 12.3	5.4 (1.0)	17.5 (1.0)	42,579 (1,937)	8,776 (769)
	Stage III (n = 5,257)	57.5 ± 11.4	3.2 (0.3)	20.5 (0.3)	35,381 (820)	12,153 (648)
	Stage IV (n = 3,344)	57.9 ± 11.4	1.0 (0.1)	22.5 (0.2)	20,907 (388)	22,426 (998)
	Unknown (n = 1,786)	59.6 ± 12.6	2.9 (0.4)	19.4 (0.4)	30,778 (1,095)	11,392 (837)
Female	Stage I+II (n = 242)	65.2 ± 13.7	5.5 (1.7)	16.0 (1.7)	37,960 (5,128)	7,468 (1,121)
	Stage III+IV(n = 510)	64.3 ± 14.3	1.9 (0.4)	20.4 (0.6)	25,331 (1,418)	13,686 (1,548)
Adenocarcinoma (n = 476)		66.4 ± 14.4	2.6 (0.6)*	15.5 (0.7)**	27,047 (2,276)	11,015 (1,172)
Squamous cell carcinoma(n = 13,088)		58.0 ± 11.7	3.4 (0.3)*	20.2 (0.3)**	34,711 (960)	10,604 (497)

^a^Lifetime costs in US dollars a exchange rate on 2015/12/31 (1 USD = 33.017 NTD) provided by Bank of Taiwan.

*p = 0.21; **p = 0.000.

## Discussion

### Lifetime risks and incidence rates: What’s new?

Our study shows that esophageal cancer in Taiwan is still a male (93.7%), and squamous cell carcinoma (90.8%) predominant disease, mostly diagnosed at advanced stages, with 44.6% and 28.3% at stages III and IV, respectively. The above findings corroborate with previous reports^2,3,13–17^. We calculated the cumulative incidence rate, which is the probability of an event occurring during a stated period of observation, if the person has not died of other diseases. Therefore, the [CIR_30–84_(‰)] can be interpreted as the lifetime risk of disease occurrence^18^. We found that the incidence of squamous esophageal cancer in males in Taiwan continuously increased with a CIR_30–84_ of 0.0146% (2008~2010) to 0.0165% (2013–2014) (Table 2). Although the same increasing trend had been reported previously^3,4,17^, they were age-standardized incidence rates and may not be easily understood by lay people. In contrast, our figures can be interpreted as the lifetime probability of developing esophageal cancer for Taiwanese males.

In addition, our findings (Table 2 and Supplementary Fig. 1) revealed a slight but consistent shift in the highest incidence rate toward a younger age-group, namely, from 60–69 y/o in 2008–2010 to 50–59 y/o in 2013–2014 in males with squamous esophageal cancer in Taiwan. Accordingly, the mean age at diagnosis of squamous esophageal cancer in Taiwanese males moved from 60 (1998~2007)^13^, and 59.1(1998–2012) years old^19^, down to 58 y/o (2008~2014) (Table 1). We conjectured that such a slight shift could be partially explained by an increased proportion of younger patients diagnosed at stage 3 in comparison to stage 4 in recent years (calendar periods of 2008–10 v.s.2011–2 and 2013–4, in Table 3). More studies are needed to explore detailed reasons for this phenomenon in the future.

### An indicator of prognosis: life expectancy

Survival rates are a measure of how many people remain alive with cancer after a certain period of time. For example, the 5- yr survival rates were 44.2% for clinical stage I (cI) of esophageal cancer patients in Taiwan; 27.5% for cII; 15.6% for cIII; and 3.4% for cIV as reported by Cheng YF *et al*.^17^. However, not everyone living with cancer is interested in hearing about statistics on survival rates. More people hope to know how long they could live with a particular type of cancer. Therefore, life expectancy (LE) would be a viable alternative when informing patients, i.e., the number of years that someone is expected to live from the date of diagnosis. In general, LE is more relevant to patients for the future planning of their daily lives. In this study, the LEs of each stage for squamous esophageal cancer in male patients in Taiwan were as follows: 8.5 yrs for stage I; 5.4 for II; 3.2 for III; 1.0 for IV; and 2.9 for unknown stage (Table 4 and Fig. 1). The LE of esophageal cancer without staging information in Taiwan (1998~2012) was 3.1 years, as reported by Wu TY *et al*.^19^, indicating very limited or no improvement in prognosis. Regarding the prognosis of different pathological types, we found that the loss-of-LE of patients with adenocarcinoma was significantly lower than that of squamous cell carcinoma, which corroborate with previous reports^20,21^. Future studies are warranted to explore the distinctions between these two pathological types.

### Indicators of societal burdens: Expected years of life loss and lifetime cost

Expected years of life lost (EYLL) is an estimate of the average years a person would have lived if he or she had not died prematurely^22^. It is therefore, a more accurate measure of premature mortality similar to deaths before 65 years old^23^. Our results showed that esophageal cancers is a disease with high premature death with >15 years of EYLL, even at the earliest stages for both males and females, 15.8 and 16.0 years of EYLL, respectively (Table 4 and Fig. 1). This implies that the unhealthy lifestyle (cigarette smoking, drinking alcohol, and betel quid chewing) of esophageal cancer patients can have a big impact on their remaining life. As stated by Ferronha l *et al*. and Kuang JJ *et al*., many cancer survivors continue smoking and /or drinking even after diagnosis, which may result in a high cumulative exposures and poor survival^24–26^. As many as 27% of esophageal cancer patients have a chances of developing metachronous second primary cancer stated by Chen MF *et al*.^13^ in a study of a Taiwan nationwide esophageal cancer database from 1998–2007. The percentage of synchronous cancers diagnosed at the same time as esophageal cancer was around 4.1–4.5%^27,28^. The percentage of antecedent tumors of esophageal cancer, i.e. esophageal cancer as a second primary, following first primary cancers of other sites in this study was 18.1% (Table 1). The EYLL of male esophageal patients with unknown staging was 19.4 in this study. Our finding was in line with that (19.1) reported by Wu TY *et al*.^19^(1998–2012). Therefore, primary prevention for avoiding known risk factors, such as cigarette smoking, drinking alcohol, and betel quid chewing, might be the best strategy to reduce morbidity and mortality from esophageal cancer^29,30^.

Another relevant indicator of societal burden is the lifetime spending for healthcare services of cancer, as summarized in Table 4. Lifetime cost for unknown staging of esophageal cancer in Taiwan male patients was US$ 30,778, which seems to be consistent with that (US$ 25,900) reported by Wu TY *et al*.^19^. In the typical studies of cost-effectiveness, researcher hope to quantify the incremental cost-effectiveness ratio (ICER), which is an overall measure representing the economic value of an intervention, compared with an alternative (comparator)^31^. An ICER is calculated by dividing the difference in total costs (incremental cost) by the difference in the chosen measure of health outcome or effect (incremental effect) to provide a ratio of “extra cost per extra unit of health effect” – for the more expensive therapy vs the alternative. Here, we considered both lifetime costs and LY (life-year) together as a cost effectiveness ratio (CER, or. Lifetime cost/LY, with a discounting rate adjusted for both numerator and denominator) for comparison across different diseases. The CER for stage IV esophageal cancer male patients was about 3.2 folds higher than that of stage I, or, US$ 22,426 v.s. 6,987 (Table 4), indicating the significance of early diagnosis. Lifetime cost mentioned here is the reimbursement costs related to the patient’s disease only, which did not include caregiver cost, transportation cost, nor costs from non–health care sectors (e.g. productivity loss, social services, and long term care, etc.), so the context of our analysis is essentially a narrower^32^. Future studies are warranted to conduct a more comprehensive assessment from a societal perspective.

In conclusion, esophageal cancer in Taiwan appears to have slightly shifted into younger ages groups and incidence is still increasing. The lifelong health impacts (lifetime risks, LEs after diagnosis, EYLL) and financial burdens (lifetime cost, & cost-effectiveness ratio) on healthcare sectors of esophageal cancer in Taiwan were quantified, which indicate the significance of developing effective strategies for prevention, early diagnosis and timely treatment for a more sustainable NHI. Future studies are warranted to explore the relative cost-effectiveness for various control strategies of this cancer.

## Materials and Methods

The study was approved by the Institutional Review Board of National Cheng Kung University Hospital (IRB number: B-ER-104–103) before commencement, which followed the ethical principles of WMA (World Medical Association)and Declaration of Helsinki (https://www.wma.net/policies-post/wma-declaration-of-helsinki-ethical-principles-for-medical-research-involving-human-subjects/). Data related to quality of life were collected after each patient provided informed consent.

### Study population and datasets

All the patients’ personal information was protected by encrypting their identification numbers, and all the analyses described below were performed in accordance with the relevant guidelines and regulations, and conducted in a secured area administered by the Health and Welfare Data Science Center, Ministry of Health and Welfare of Taiwan (https://dep.mohw.gov.tw/DOS/sp-GS-113.html?Query and https://dep.mohw.gov.tw/DOS/sp-GS-113.html?Query). Only summary tables could be brought out after verification by the officials to assure that there was no leakage of personal information.

**Figure 2:**
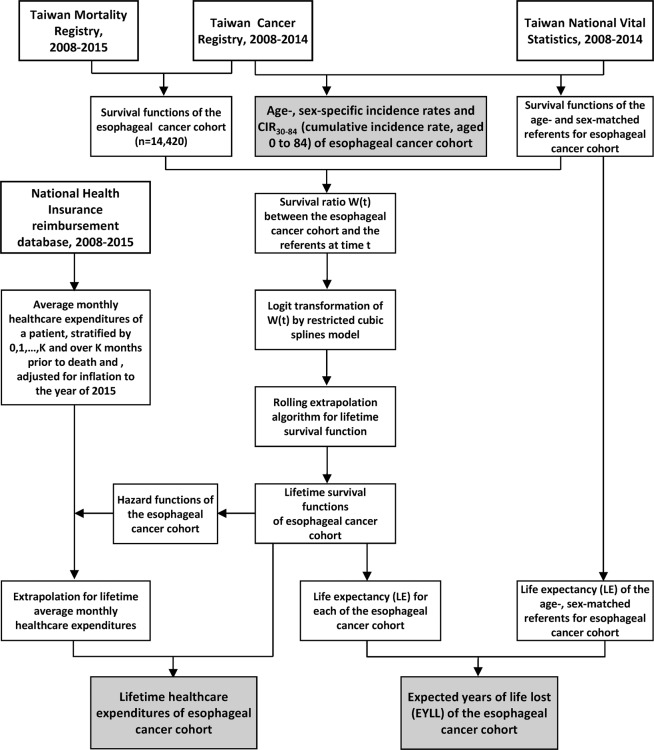
Flow diagram of the establishment of the Taiwan esophageal cancer cohort and retrieval of relevant data for the estimation of lifetime risks and outcomes.

Esophageal cancer included ICD-10-CM codes: C153, C154, C155, C158, C159, and ICD-O-3 morphology code: 8050/03~8089/3(squamous cell carcinoma), 8140/3(adenocarcinoma), and others. The stages of all esophageal cancer patients were stratified by pathological stage. If there was no pathologic staging information, the clinical stage was used. We interlinked the following 3 datasets, as summarized in Fig. 2: The Taiwan Cancer Registry from 2008 to 2014, the reimbursement database of the NHI system (2008–2015), and the Taiwan Mortality Registry (2008–2015) to ascertain every patient’s current survival state. A total of 14,420 esophageal cancer patients with histopathological proof aged between 30 and 99 years old were retrieved for patients’ demographics, date of diagnosis, cancer site, histology, and health care expenditure covered by the NHI. As cancer is a catastrophic illness, all costs of outpatient clinic visits and hospitalization, including laboratory and imaging studies and prescription medicines were covered by the NHI and waived from copayment. If patients were still alive at the end of December 2015, they were censored.

### Estimation of lifetime risk by Cumulative incidence Rate (CIR)

We calculated the age- and sex-specific incidence rates and CIR for the esophageal cancers. The numerators were the age-, sex-specific number of new cases from the Taiwan Cancer Registry for 2008–2010, 2011–2012, 2013–2014, and the corresponding populations at risk were abstracted from vital statistics as the denominators. The CIR was calculated from ages 30 to 84 (CIR_30–84_) to estimate the lifetime risk of this specific cancer. The CIR_30–84_ was calculated from 2008 to 2014 to determine the trends in changes of lifetime risks. The formula is as follows^33^:

$${{\rm{CIR}}}_{30\mbox{--}84}=1-\exp \,(\,-\,\sum ({{\rm{IR}}}_{{\rm{i}}})\,({\Delta {\rm{t}}}_{{\rm{i}}})),\,{\rm{where}}\,{i}=30\mbox{--}34,35\mbox{--}39,\ldots ,80\mbox{--}{84}^{{\rm{i}}}$$

where IR_i_ is the incidence rate for the i-th age group and Δt_i_ = 5 year age range.

### Estimation of lifetime survival function, LE after diagnosis, and EYLL

The survival status of each esophageal cancer patient was acquired from the National Mortality Registry during 2008–2015. The survival functions were calculated with the Kaplan–Meier estimation method until the end of follow-up.

**Figure 3:**
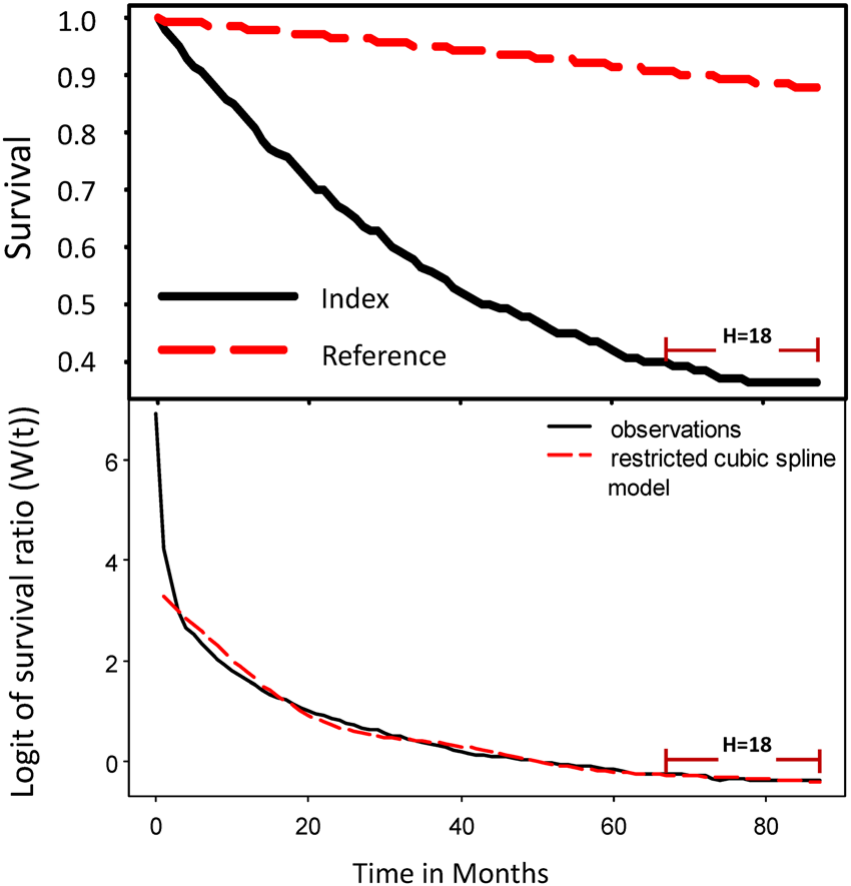
The upper panel shows survival functions of the index cohort (esophageal cancer, the blackish curve) and sex, age, and calendar-year matched reference cohort (the reddish dashed curve) simulated from vital statistics. The relative survival *W*(*t*) is defined as the ratio of survival functions between the index group and reference population, namely, S_1_(t)/S_0_(t). The lower panel shows the fitted restricted cubic spline model of the logit of *W*(*t*) up to the end of follow-up, which fits well with the actual survival curve and the model of the last 18 months is linear (indicated as H = 18). With this part of the curve, we extrapolated one month by assuming that it would be still linear within the next one month. Then, we refitted the model again by adopting the extrapolated one month as the observed one and further rolling over another one more month, etc.

The extrapolation of the survival function was estimated using a rolling extrapolation algorithm, aided by the age- and sex-matched referents simulated from the National Vital Statistics life tables, as detailed in previous literature^19,34^. The method could be briefly summarized as follows: First, for every patient in our cohort, we generated the age-, sex-, and calendar year of diagnosis-matched referents based on the life tables of Taiwan during the study period and estimated the Kaplan-Meier’s lifetime survival function. Namely, we applied the hazard rates of the life table of the same calendar year for each individual patient to simulate the lifetime survival function of matched referents after the corresponding age, as all the blackish curves of Fig. 1 indicated. Second, we calculated the survival ratio between the cohorts of esophageal cancer and the referents at each time point and performed logit transformation of this ratio. Third, we fitted the logit-transformed relative survival curve into a restricted cubic splines model to extrapolate the survival function for one extended month by assuming that the fitted curve would be linear within the extrapolated one month, as shown in Fig. 3. By repeatedly perform the above procedure month-by-month (i.e., rolling over), we extrapolated the survival function of patients with esophageal cancer to their lifetimes, as shown in Fig. 1 after indicated end of follow-up. Because LE is the summation of the area under the lifetime survival curve, we were able to obtain the EYLL by comparing the LEs between the cancer cohort and that of the correspondingly matched referents, namely, the loss of life expectancy. The iSQoL 2 software was used to compute these estimations^35^.

### Estimation of average monthly cost and its extrapolation to lifetime

As mentioned above, all costs of outpatient clinic visits and hospitalization of esophageal cancer patient were covered by the NHI. Therefore, our reimbursement data for this cancer was very comprehensive.

We summed up all the reimbursement costs for every esophageal cancer case and calculated the monthly average cost after diagnosis. The estimated lifetime cost was quantified by adding the product of monthly survival rates and monthly mean costs^34^. Briefly, since we assumed that the medical expenditures spent near mortality increased when the patient with esophageal cancer approached the end of life, we stratified the healthcare costs of those who died during the study period and those who were censored. We then analyzed the real data of those who died during the follow-up period to determine the specific month of when such costs began to increase approaching death and the increasing trend over time. Then, the monthly costs at time *t* beyond the maximum follow-up time was calculated by a weighted sum of costs who were alive, costs who were near deaths, and costs who died on that month, where weights were the corresponding probabilities of survival at time *t*, death at a specific number of months after *t*, and death at time *t*, respectively^34^.

## Supplementary information

Supplementary Figure 1.
