# Supplementary material for: Lifetime risks, expected years of life lost, and cost-per-life year of esophageal cancer in Taiwan
Source: Sci Rep. 2020 Feb 28;10:3722. doi: 10.1038/s41598-020-60701-3 (PMC7048720; doi:10.1038/s41598-020-60701-3)

Lifetime risks, expected years of life lost, and cost-per-life year of esophageal cancer in Taiwan

Wu-Wei Lai, MD[1,] Chia-Ni Lin MS[2], Chao-Chun Chang, MD[1], Jung-Der Wang, MD, ScD [2, 3*]

Supplementary figure 1:

Incidence rate of esophageal cancer in male stratified by pathology type and year-period

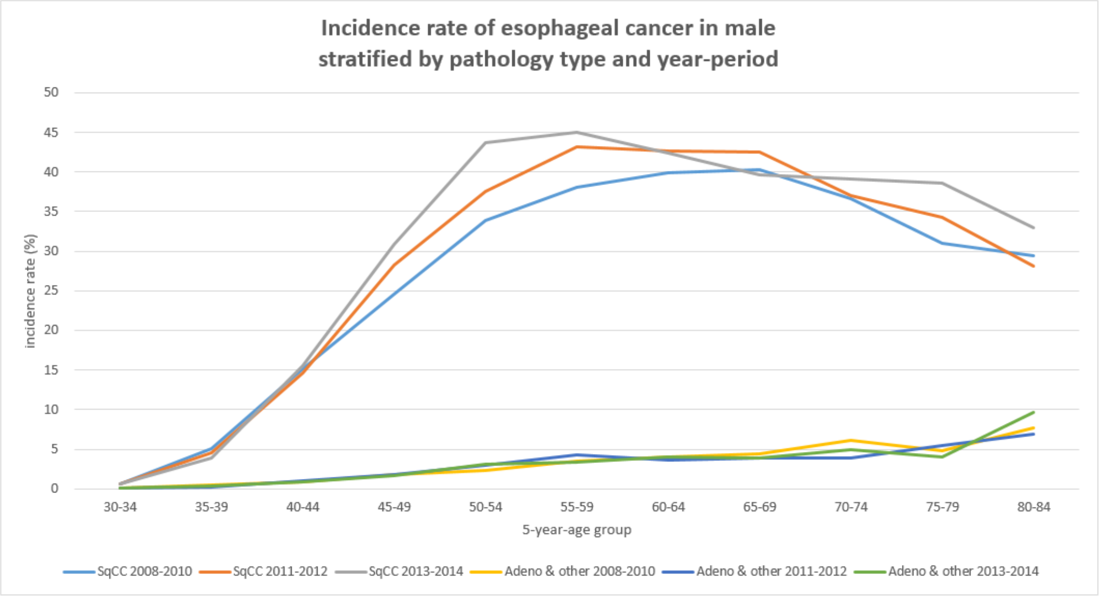

Supplement: Supplementary file 1 — Supplementary Figure 1. [file 41598_2020_60701_MOESM1_ESM.pdf]
